# A narrative review: key evidence from meta-analyses in nuclear medicine

**DOI:** 10.1007/s12149-026-02201-4

**Published:** 2026-04-07

**Authors:** Yuji Nakamoto, Mitsuaki Tatsumi, Ryuichi Nishii, Katsuhiko Kato, Yu Iwabuchi, Atsutaka Okizaki

**Affiliations:** 1 Department of Diagnostic Imaging and Nuclear Medicine, Graduate School of Medicine, Kyoto University, 54 Shogoinkawahara-cho, Sakyo-ku, Kyoto, 606-8507 Japan; 2 Department of Diagnostic Radiology, Juntendo University Graduate School of Medicine, 2-1-1 Hongo, Bunkyo-ku, Tokyo, 113-8421 Japan; 3 Biomedical Imaging Sciences, Department of Integrated Health Sciences, Nagoya University Graduate School of Medicine, 1-1-20 Daiko-Minami, Higashi-ku, Nagoya, 461-8673 Japan; 4 Department of Radiology, Nagoya University Graduate School of Medicine, 65 Tsurumaicho, Showa-ku, Nagoya, 466-8550 Japan; 5 Department of Radiology, Keio University School of Medicine, 35 Shinanomachi, Shinjuku-ku, Tokyo, 160-8582 Japan; 6 Department of Radiology, Asahikawa Medical University, 2-1-1-1 Midorigaoka-higashi, Asahikawa, 078-8510 Japan

**Keywords:** Nuclear medicine, PET, Meta-analysis, Systematic review

## Abstract

Recent progress in nuclear medicine has been driven by continuous advances in radiotracers, imaging technologies, and radiopharmaceutical therapies, leading to an expansion of clinical applications supported by an increasing body of evidence. In particular, systematic reviews and meta-analyses have played an important role in quantitatively evaluating the clinical value of these developments. This narrative review provides an overview of recent meta-analytic evidence in nuclear medicine, with a primary focus on oncologic applications, while briefly addressing developments in cardiology and neurology. In oncology, positron emission tomography (PET)/computed tomography (CT) using fluorodeoxyglucose (FDG) has become an established imaging modality for post-treatment assessment across various malignancies. Meta-analyses indicate that volumetric metabolic parameters, such as metabolic tumor volume and total lesion glycolysis, are more consistently associated with clinical outcomes than conventional single-voxel metrics. In addition, the interim and post-therapy metabolic response, assessed by FDG PET/CT, provides clinically relevant prognostic information and may contribute to treatment monitoring. Beyond FDG, tumor-targeted PET tracers, including prostate-specific membrane antigen and somatostatin receptor ligands, have demonstrated improved diagnostic performance in specific malignancies, and emerging tracers such as fibroblast activation protein inhibitors represent promising future directions. Radiopharmaceutical therapy has also shown notable progress, with meta-analytic evidence supporting the effectiveness and acceptable safety of peptide receptor radionuclide therapy for neuroendocrine tumors, as well as for advanced prostate cancer. In nuclear cardiology and neurology, advances in PET and single photon emission CT technologies have enabled more precise disease characterization, although their clinical roles continue to evolve. Overall, accumulating meta-analytic evidence supports the growing clinical importance of nuclear medicine. Continued methodological refinement and further evidence generation will be essential to optimize patient management in routine clinical practice.

## Introduction

In contemporary medicine, the advent of innovative technologies has driven substantial advancement across numerous disciplines. Nuclear medicine is no exception; the field has witnessed the continuous emergence of novel radiotracers and imaging devices, several of which have gained acceptance and widespread clinical application. A number of systematic reviews and meta-analyses have already evaluated the diagnostic and therapeutic performance of these technological developments.

In this narrative review, we selected and discussed meta-analytic studies published mainly in the past decade to provide an informed overview of the latest progress in nuclear medicine, with a particular focus on applications in oncology, cardiology, and neurology.

## FDG PET/CT in oncology

Positron emission tomography (PET) /computed tomography (CT) using fluorodeoxyglucose (FDG) is an important modality in oncologic practice, with an expanding role in treatment response assessment and prognostication. Since the advent of molecularly targeted drugs and immune checkpoint inhibitors (ICIs), the usefulness of FDG PET for assessing treatment response has been reported with increasing frequency across various malignancies. However, in Japan, FDG PET for treatment response assessment is currently covered by insurance only for malignant lymphoma. We summarize meta-analyses published between 2015 and 2024, focusing on treatment response assessment and prognostication in head and neck and thoracic malignancies.

*For head and neck cancer*, PET/CT has become a central component of post-treatment evaluation and prognostication in head and neck squamous cell carcinoma. Recent systematic reviews and meta-analyses provide complementary insights into its diagnostic accuracy for detecting residual nodal disease, the prognostic value of baseline metabolic parameters, and the predictive significance of intra-therapy and post-therapy metabolic response. A comprehensive meta-analysis by Helsen et al. evaluated 20 studies comprising 1,293 patients who underwent FDG PET/CT within 6 months after chemoradiotherapy (CRT) [1]. The pooled sensitivity and specificity for detecting residual nodal disease were 85% and 93%, respectively, with an area under the curve (AUC) of 0.94. The negative predictive value (NPV) was particularly high—98% at a pretest probability of 10%—supporting PET-guided surveillance strategies and selective omission of planned neck dissection. In contrast, the positive predictive value (PPV) remained modest (58% at 10% prevalence), reflecting the difficulty of distinguishing post-treatment inflammation from viable tumor.

Beyond diagnostic utility, FDG PET-derived metabolic parameters have been extensively investigated as prognostic biomarkers. In a meta-analysis of 25 studies including 2,223 patients, Bonomo et al. demonstrated that pretreatment metabolic tumor volume (MTV) was consistently associated with adverse outcomes [2]. High MTV predicted inferior overall survival (OS) (relative risk < RR> 1.86), progression-free survival (PFS) (RR 1.81), and locoregional control (RR 3.49). These associations remained robust despite heterogeneity in segmentation thresholds, volume of interest (VOI) definitions, and PET acquisition techniques. In contrast, maximum standardized uptake value (SUVmax) showed no significant association with survival endpoints, highlighting the limitations of single voxel metrics in biologically heterogeneous tumors. Total lesion glycolysis (TLG), although less frequently reported, demonstrated similar trends to MTV. These findings support the incorporation of volumetric metabolic parameters into risk stratification and potentially adaptive treatment strategies.

Sheikhbahaei et al. summarized data from 26 studies evaluating the prognostic significance of intra-therapy or post-therapy FDG PET/CT [3]. A positive PET result—whether during treatment or after completion—was strongly associated with inferior outcomes. Pooled hazard ratios (HR) were 3.55 for OS and 4.73 for event-free survival, indicating that metabolic nonresponse is a powerful predictor of early treatment failure.

Risk ratio analyses further demonstrated that a positive PET increased 2-year mortality risk more than six-fold (risk ratio 6.19), with persistent significance at 3–5 years (risk ratio 2.42). Recurrence or progression risk was also markedly elevated, with pooled risk ratios of 3.57 at 2 years and 3.14 at 3–5 years. PET performed ≥ 12 weeks after CRT showed stronger prognostic discrimination than earlier imaging, consistent with the temporal evolution of post-treatment inflammation. These findings support metabolic response as a surrogate endpoint in clinical trials and a tool for identifying candidates for early salvage interventions.

For breast cancer, FDG-based molecular imaging has emerged as a critical tool for evaluating therapeutic response during and after neoadjuvant chemotherapy (NAC). Three recent systematic reviews and meta-analyses provide insights into the diagnostic accuracy of FDG PET/CT for predicting pathological response, the incremental value of hybrid PET/MRI, and the prognostic significance of metabolic response parameters.

Tian et al. reviewed 22 studies involving 1,119 patients to evaluate FDG PET/CT performance in predicting pathological complete response (pCR) or responder status during early NAC [4]. Using change in SUVmax (ΔSUVmax) as the primary metric, pooled sensitivity and specificity were 81.9% and 79.3%, respectively, with a diagnostic odds ratio of 17.35. Studies using ΔSUVmax ≥ 50% tended to show higher sensitivity, aligning with European Organisation for Research and Treatment of Cancer criteria (EORTC). However, heterogeneity in pathological definitions, region of interest segmentation, and NAC regimens limited comparability. Ghanikolahloo et al. evaluated six studies (*n* = 239) assessing integrated FDG PET/magnetic resonance imaging (PET/MRI) for predicting pCR [5]. Pooled sensitivity and specificity were 91% and 62%, outperforming MRI alone (78% and 56%). PET/MRI also demonstrated higher PPV (92%) and comparable NPV (63%). These findings highlight the synergistic value of combining metabolic and high-resolution morphological information, particularly in heterogeneous tumors. Han and Choi analyzed 17 studies (*n* = 1,279) evaluating metabolic response during or after NAC [6]. The most widely used parameter was percentage change in SUVmax (%ΔSUVmax). For interim PET, pooled HRs for disease-free survival (DFS) and OS were 0.21 and 0.20, indicating a 70–80% reduction in recurrence or death among metabolic responders.

For esophageal cancer, the interim FDG PET has emerged as a key biomarker for assessing early metabolic response during neoadjuvant CRT. A systematic review and meta-analysis of 11 studies (695 patients) demonstrated high pooled sensitivity (80%) but modest specificity (54%) for predicting pathological response [7]. The hierarchical summary receiver operating characteristic (HSROC) AUC was 0.64, indicating moderate discriminative performance.

For lung cancer, a systematic review and meta-analysis evaluated the prognostic significance of baseline FDG PET/CT metabolic parameters in advanced or metastatic non-small cell lung cancer treated with ICIs [8]. Thirteen studies assessed SUVmax, mean standardized uptake value (SUVmean), MTV, and TLG as predictors of survival outcomes. SUVmax and SUVmean demonstrated limited prognostic value, with pooled HRs near unity for both OS and PFS. In contrast, volumetric parameters showed strong and consistent associations with prognosis. High MTV correlated with inferior OS (HR 2.10) and PFS (HR 1.50), with similar trends for TLG. Subgroup analyses, which stratified patients by treatment line, confirmed that MTV remained a consistent prognostic marker. This reliability was most evident in first-line ICI therapy, where high baseline MTV was strongly associated with poor OS (HR 1.97).

## Promising tracers other than FDG

Beyond FDG, several tumor-seeking PET radiotracers have expanded the diagnostic scope of nuclear oncology. Among them, prostate-specific membrane antigen (PSMA)–targeted imaging and somatostatin receptor (SSTR)–based imaging represent the most established and clinically impactful approaches, whereas newer tracers such as fibroblast activation protein inhibitor (FAPI) PET illustrate emerging directions in oncologic imaging.

PSMA PET has become a central imaging modality for prostate cancer, particularly in the setting of biochemical recurrence (BCR). An updated systematic review and meta-analysis by Perera et al. evaluated 65 studies comprising more than 4,700 patients undergoing ^68^Ga-PSMA PET across different disease stages [9]. The pooled detection rate increased with prostate-specific antigen (PSA) levels, ranging from approximately 33% at PSA < 0.2 ng/mL to over 90% at PSA ≥ 2.0 ng/mL, thereby quantitatively clarifying the relationship between PSA levels and lesion detection. This comprehensive analysis consolidated PSMA PET as the reference molecular imaging technique for prostate cancer and has served as a significant evidence base for subsequent clinical guidelines and consensus recommendations. The diagnostic efficacy of ^68^Ga-PSMA-11 PET/CT was further substantiated by Kimura et al., whose meta-analysis of 462 patients revealed exceptional accuracy in identifying metastatic sites before salvage lymph node dissection [10]. Lesion-based results yielded a pooled sensitivity and specificity of 84% and 97%, respectively. The clinical significance of these findings is bolstered by the use of histopathological validation as the gold standard, confirming the modality’s high positive predictive value and overall robustness in the surgical planning setting.

More recently, the development of ^18^F-labeled PSMA tracers has introduced additional practical and technical advantages, including a longer radionuclide half-life and improved spatial resolution. A systematic review and meta-analysis by Yang et al. included 16 studies with 1,162 patients with BCR. They demonstrated pooled sensitivities and specificities of approximately 93% and 94%, respectively, for ^18^F-labeled PSMA PET/CT [11]. These results confirmed diagnostic performance comparable to, or exceeding, that of ^68^Ga-labeled agents, highlighting an ongoing transition toward ^18^F-based PSMA imaging and illustrating the evolution of PSMA PET from an emerging technique to a globally standardized diagnostic tool over the past decade.

The introduction of SSTR PET has fundamentally transformed the diagnostic paradigm for neuroendocrine tumors (NETs). In a landmark systematic review and meta-analysis, Deppen et al. evaluated 10 studies involving 465 patients, demonstrating that ^68^Ga-DOTATATE PET/CT achieved a pooled sensitivity of 90.9% and a pooled specificity of 90.6% [12]. These results confirmed that ^68^Ga-DOTATATE significantly outperforms conventional imaging and ^111^In-DTPA-octreotide scintigraphy, particularly for pulmonary and gastroenteropancreatic NETs. This pivotal study underscored a decisive generational shift from conventional scintigraphy to PET-based molecular imaging, establishing SSTR PET as the superior diagnostic standard in clinical practice.

Subsequently, Bauckneht et al. conducted a systematic review and meta-analysis focusing on the detection of unknown primary NETs, incorporating data from 14 studies involving more than 400 patients [13]. The pooled detection rate of SSTR PET/CT for identifying the primary tumor was approximately 61%, with particularly high performance in well-differentiated and early-stage disease. These findings emphasize that SSTR PET is not merely a staging modality but a critical diagnostic tool that directly informs therapeutic decision-making, including eligibility for peptide receptor radionuclide therapy (PRRT), thereby linking diagnostic imaging to personalized treatment strategies.

As for the FAPI, two highly significant studies provide a comprehensive understanding of the clinical utility of FAPI PET imaging in oncology. Wass et al. conducted a broad systematic review and meta-analysis of 39 studies involving 1,259 patients, revealing that FAPI PET/CT exhibits a very high pooled sensitivity of 99% (95% CI: 97–100) for detecting primary lesions [14]. Furthermore, it demonstrated high diagnostic performance in detecting lymph node (sensitivity of 91%) and distant metastases (sensitivity of 99%), highlighting its potential advantages over conventional FDG PET in several oncologic settings. Building upon such solid data, Kirienko et al. conducted an umbrella review that synthesized evidence by meticulously selecting 64 high-quality meta-analyses from an initial pool of 449 records [15]. While this review provides a broad comparative framework for various PET radiotracers, the available evidence for FAPI remains heterogeneous and is currently more robust in selected malignancies. These findings suggest that, although promising, the clinical role of FAPI PET is still evolving within the broader landscape of personalized oncological imaging. The key pooled sensitivity and specificity estimates for representative oncologic PET applications are summarized in Table 1.

## Recent topics in nuclear medicine treatments

In nuclear medicine, various radiopharmaceutical therapies are currently available and the indications for radioligand therapy are expanding. Here, we describe recent systematic reviews and meta-analyses of peptide receptor radionuclide therapy (PRRT, with lutetium-177 [^177^Lu] DOTATATE) for *NETs*, ^177^Lu-labeled *PSMA*-targeted radioligand therapy in metastatic castration-resistant prostate cancer, and ^225^Ac-PSMA-617-targeted alpha therapy for the treatment of metastatic castration-resistant prostate cancer (mCRPC).

Kaderli et al. systematically reviewed and compared the effectiveness of various systemic treatments for advanced NETs [16]. In light of the increasing incidence of NETs and the growing number of available therapies, they conducted a network meta-analysis to evaluate the relative efficacy and safety of these therapies. They analyzed data from 30 randomized clinical trials involving a total of 3,895 patients. The primary endpoint was *PFS*, and the secondary endpoints included overall survival (OS) and serious adverse events. Therapies included everolimus, sunitinib, PRRT, somatostatin analogs, chemotherapy, interferon, and placebo. PRRT (^177^Lu-DOTATATE) resulted in the most significant improvement in PFS as compared to placebo. Everolimus and sunitinib also showed benefits, particularly for pancreatic NETs. In terms of safety, PRRT is associated with relatively few serious adverse events, whereas chemotherapy and interferons have higher toxicity profiles. PRRT (^177^Lu-DOTATATE plus somatostatin analog) showed the strongest PFS benefit in GI-NETs in the network meta-analysis. The study suggests that combination therapies may be more effective overall, while safety profiles varied considerably across treatments; therefore, treatment selection should balance efficacy and toxicity.

In a systematic review and meta-analysis, Satapathy et al. evaluated the therapeutic efficacy and safety of ^225^Ac-PSMA radioligand therapy in mCRPC patients who had exhausted conventional treatments [17]. The authors analyzed 10 studies comprising 256 patients with mCRPC who were treated with ^225^Ac-PSMA. The primary outcomes were prostate-specific antigen (PSA) response, OS, and treatment-related toxicities. *Overall*, 62.8% of patients had a PSA decline ≥ 50%, indicating a strong biochemical response. Molecular imaging response on Gallium-68 PSMA PET/CT was observed in 74% of patients. The pooled median OS was 12.8 months, which was notable, given the advanced disease state of the included patients. This therapy showed promising efficacy even in populations that had been heavily pretreated, including those previously treated with chemotherapy or other PSMA-targeting agents. In terms of safety, xerostomia (dry mouth) was the most frequently reported side effect, followed by hematological toxicity; however, severe adverse events were relatively uncommon. Satapathy et al. concluded that ^*225*^Ac-PSMA therapy is a highly promising option for mCRPC, with substantial therapeutic benefits and a manageable safety profile. However, the authors emphasize the need for larger prospective randomized trials to establish its role and optimize dosing strategies and patient selection further.

In another systematic review and meta-analysis, Ballal et al. assessed the efficacy and safety of ^225^Ac-labeled PSMA-617 (^225^Ac-PSMA-617)- targeted alpha therapy in patients with mCRPC [18]. They reviewed three studies involving 141 patients with progressive mCRPC who were often refractory to standard treatments, including chemotherapy and androgen receptor-targeted agents. The primary endpoint was biochemical response, particularly PSA decline, whereas the secondary endpoints were OS and treatment-related toxicities. They found that 83% of patients experienced some degree of PSA decline, while 59% had a ≥ 50% PSA decline, demonstrating a strong therapeutic response. The pooled median OS was approximately 17 months. In terms of safety, xerostomia was the most common adverse effect associated with salivary gland uptake. Hematologic and renal toxicities were observed, but were generally mild and manageable. Importantly, the treatment showed benefits even in heavily pretreated patients with advanced disease, suggesting its potential as a salvage therapy. The authors concluded that ^225^Ac-PSMA-617 is a promising and effective treatment option for mCRPC, offering meaningful clinical benefits with an acceptable safety profile. They emphasized the need for large-scale randomized controlled trials to validate these findings and refine patient selection and dosing protocols further.

Sadaghiani et al. reported a systematic review and meta-analysis evaluating the clinical effectiveness and safety of ^177^Lu-PSMA radioligand therapy in patients with mCRPC [19]. The authors conducted a comprehensive literature search to identify studies reporting the treatment outcomes and toxicities associated with ^177^Lu-PSMA therapy. Twenty-four studies, encompassing 1,192 patients, met the inclusion criteria. A pooled analysis demonstrated that approximately 44% of the patients achieved a PSA decline of at least 50%, indicating a significant biochemical response. Many patients also experienced symptomatic relief, including pain reduction and improved quality of life. Regarding safety, hematologic toxicities, such as anemia and thrombocytopenia were the most frequent adverse events, whereas non-hematologic effects, such as xerostomia and mild nausea, were commonly reported but were generally mild. Severe toxicities (grades 3–4) were relatively infrequent, and no treatment-related deaths were observed. These results suggested that ^177^Lu-PSMA radioligand therapy offers meaningful therapeutic benefits and has an acceptable safety profile in patients with advanced treatment-resistant prostate cancer. However, the authors emphasized that most included studies were retrospective and heterogeneous in design, dosing, and follow-up duration. They concluded that further large-scale prospective randomized controlled trials are necessary to confirm the efficacy and to optimize the use of ^177^Lu-PSMA radioligand therapy in mCRPC management.

## Nuclear cardiology

Remarkable advances have been made in cardiac nuclear imaging over the past decade. These advances have provided non-invasive insights into coronary perfusion, myocardial inflammation, and cardiac amyloid deposition through tracer kinetics and advanced PET or SPECT detector technologies. Recent systematic reviews and meta-analyses have emphasized the growing clinical importance and diagnostic accuracy of new nuclear imaging techniques, such as PET and dynamic cadmium–zinc–telluride (CZT) SPECT, in the diagnosis of various cardiac conditions.

The recently introduced CZT camera delivers higher resolution and shorter acquisition times [20, 21]. It improves overall SPECT performance and decreases radiation dose. Importantly, CZT cameras enable quantification of myocardial blood flow (MBF) and myocardial flow reserve (MFR), providing a more accurate assessment of multivessel, left main, and microvascular disease. Panjer et al. evaluated the diagnostic accuracy of dynamic CZT SPECT in coronary artery disease (CAD) compared to quantitative CAG, FFR, and PET as reference [22]. They included mostly single-center, prospective cohort studies (88.9%) which were published between 2015 and 2020. They encompassed a total of 421 patients from nine studies. They demonstrated that a pooled analysis using a bivariate model yielded sensitivities and specificities of 79% (95% confidence interval [CI], 73 to 85) and 85% (95% CI, 74 to 92), respectively, for CZT-SPECT assessment against both PET and FFR comparators. This suggests that dynamic CZT-SPECT could be used as an alternative to the current gold standards for functional and quantitative anatomical assessments in the identification of hemodynamically significant coronary stenosis. This capability stems from the high temporal resolution and count sensitivity of CZT detectors, which enable dynamic acquisition and kinetic modelling. These findings position CZT-SPECT not merely as a functional extension of traditional perfusion imaging but as a quantitative tool capable of assessing microvascular dysfunction, thereby bridging the gap between anatomical and physiological assessment of CAD.

**Table 1:** Summary of diagnostic performance in oncology

Application	No. of studies	No. of patients	Pooled sensitivity	Pooled specificity
Detection of residual nodal disease after chemoradiotherapy in head and neck squamous cell carcinoma using FDG PET/CT [1]	20	1,293	85%	93%
Prediction of pathological complete response during early neoadjuvant chemotherapy in breast cancer using FDG PET/CT [4]	22	1,119	81.9%	79.3%
Prediction of pathological complete response after neoadjuvant chemotherapy in breast cancer using FDG PET/MRI [5]	6	239	91%	62%
Prediction of pathological response during neoadjuvant chemoradiotherapy in esophageal cancer using FDG PET [7]	11	695	80%	54%
*Detection of nodal metastasis before salvage lymph node dissection using* ^*68*^*Ga-PSMA-11 PET/CT *[10]	*14*	*462*	*84%*	*97%*
Detection of biochemical recurrence of prostate cancer using ¹⁸F-PSMA PET/CT [11]	*16*	*1162*	*93%*	*94%*
Detection of pulmonary and gastroenteropancreatic neuroendocrine tumors using ⁶⁸Ga-DOTATATE PET [12]	10*	*465*	*91%*	91%

* For estimating specificity, a total of five studies were assessed

Cardiac sarcoidosis is a serious, potentially life-threatening condition and the leading cause of death in sarcoidosis patients in Japan. In an autopsy-based study of 320 cases, cardiac sarcoidosis accounted for the highest proportion of sarcoidosis-related deaths, occurring in 150 of 194 cases (46.9%) [23]. Notably, a recent population-based study reported that a total of 3,094 sarcoidosis-associated deaths were recorded over the 20-year period from 2001 to 2020, with the overall age-adjusted sarcoidosis mortality rate increasing 2.46-fold during the study period [24]. Mortality is primarily attributed to arrhythmias and cardiomyopathy induced by the disease process. In cardiac sarcoidosis, a recent systematic review of risk stratification using ^18^F-FDG PET underscores the prognostic and diagnostic value of metabolic imaging in detecting active granulomatous inflammation. Kafil et al. performed a systematic review and meta-analysis looking at the prognostic value of FDG PET imaging in patients with cardiac sarcoidosis [25]. They identified 55 studies comprising 5,250 patients and conducted six meta-analyses to explore the predictive capabilities of multiple cardiac PET parameters. Their study demonstrated that factors associated with major adverse cardiac events showed the following: the combination of abnormal FDG uptake and perfusion defect, which had an OR of 2.86 (95% CI: 1.74–4.71; *P <* 0.0001); abnormal perfusion or FDG uptake, which had an OR of 2.69 (95% CI: 1.67–4.33); abnormal FDG uptake, which had an OR of 2.61 (95% CI: 1.51–4.50); focal abnormal right ventricular uptake, which had an OR of 6.27 (95% CI: 3.19–12.32; *P <* 0.00001); and a lack of response to immunosuppression on serial PET, which had an OR of 8.43 (95% CI: 3.25–21.85; *P <* 0.0001). These results suggest that multiple cardiac PET parameters can be used to stratify risk in patients with cardiac sarcoidosis. In particular, a focal right ventricular uptake and an absence of response to immunosuppressive therapy on serial PET imaging were predictive of major adverse cardiac events.

Nevertheless, diagnostic performance remains critically dependent on patient preparation protocols, as demonstrated by another meta-analysis focusing on the impact of preparation on FDG PET in cardiac sarcoidosis. Tang et al. aimed to assess the effect of different patient preparations on the diagnostic accuracy of FDG PET/CT in cardiac sarcoidosis [26]. Their meta-analysis encompassed 16 studies (559 patients) from 2003 to 2014. Subgroup analysis used information from 15 studies (*n* = 522), based on the availability of information relating to the defined subgroups. The results showed that FDG PET had an overall sensitivity of 81% (95% CI, 76–86) and a specificity of 82% (95% CI, 77–86). Meta-regression analysis revealed that the diagnostic odds ratio was significantly influenced by fasting time and heparin administration before scanning (*P* = 0.01 and 0.02, respectively), though not by a high-fat, low-carbohydrate diet (*P* = 0.17). Their work reveals substantial variability in sensitivity and specificity depending on dietary manipulation and fasting regimens. Standardized patient preparation is a key factor in achieving diagnostic accuracy. It improves image quality by suppressing physiological myocardial glucose uptake, thereby enhancing the contrast between lesions and the background.

In contrast to the inflammatory phenotype of sarcoidosis, transthyretin cardiac amyloidosis (ATTR-CA) represents a chronic infiltrative cardiomyopathy in which bone scan tracers have proven uniquely effective for noninvasive diagnosis. Zhao et al. systematically analyzed the performance of gamma-emitting and positron-emitting bone tracers on diagnosis and differentiation of TTR-CA in their meta-analysis [27]. Fourteen articles were included in the systematic review, and 9 in the meta-analysis. The results showed that the pooled sensitivity was 97% (95% confidence interval [95% CI] 85–99) and the specificity was 92% (95% CI 82–96). The pooled positive and negative likelihood ratios were 11.49 (95% CI 5.07–26.0) and 0.03 (95% CI 0.01–0.18), respectively. The diagnostic odds ratio was 341 (95% CI 53–2194), and the area under the receiver operating characteristic curve was 0.96 (95% CI 0.94–0.97). Their findings suggest that the bone radiotracer is a valuable, non-invasive approach that provides a high level of accuracy in diagnosing TTR-CA. It also plays a modest role in distinguishing TTR-CA from immunoglobulin amyloid light-chain cardiac amyloidosis (AL-CA). This indicates that bone tracer scintigraphy could be used as the primary diagnostic method, potentially eliminating the need for an endomyocardial biopsy. Moreover, the study provides comparative insights into the relative performance of individual tracers, suggesting that ^99m^Tc-HMDP may be more accurate than ^99m^Tc-PYP, ^99m^Tc-DPD, and ^18^F-NaF in the TTR-CA detecting process. These results have reinforced the integration of bone scintigraphy into diagnostic algorithms endorsed by international consensus guidelines. The pooled sensitivity and specificity reported for major nuclear cardiology applications are summarized in Table 2.

**Table 2:** Summary of diagnostic performance in cardiology

Application	No. of studies	No. of patients	Pooled sensitivity	Pooled specificity
Diagnosis of coronary artery disease using dynamic CZT-SPECT perfusion imaging [22]	9	421	79%	85%
Diagnosis of cardiac sarcoidosis using FDG PET/CT [26]	15	522	81%	82%
Diagnosis of transthyretin cardiac amyloidosis using bone scintigraphy tracers [27]	9	310 ATTR-CA / 186 controls	97%	92%

CZT, cadmium–zinc–telluride; ATTR-CA, transthyretin cardiac amyloidosis

## Nuclear neurology

Several disease-modifying drugs for Alzheimer’s disease (AD) have been made available in Japan, depending on the results of some clinical trials [28, 29]. Therefore, precise diagnosis of AD in the earlier stage may be more important. *Yeo* et al. performed a pooled analysis of ^18^F-labeled amyloid imaging in AD [30]. Nineteen studies (10 with Florbetapir, 6 with Florbetaben, and 3 with Flutemetamol), investigating 682 patients with AD and 639 patients with non-AD were assessed. The calculations were performed using the DerSimonian-Laird random-effects model. The pooled weighted sensitivity, specificity, positive likelihood ratio (PLR), negative likelihood ratio (NLR), and diagnostic odds ratio (DOR) in differentiating AD patients from normal controls were 89.6%, 87.2%, 7.90, 0.108, and 91.7 with Florbetapir, 89.3%, 87.6%, 6.06, 0.141, and 69.9 with Florbetaben, respectively. There were insufficient data to complete analyses for flutemetamol. The authors concluded that the results suggest favorable sensitivity and specificity of amyloid imaging with ^18^F-labeled tracers in AD.

Parkinson’s disease (PD) is a common chronic neurodegenerative disease, and the number of PD patients is expected to 12 million by 2040 [31]. Histopathological evaluation with biopsy is the gold standard to make a diagnosis of PD; however, a biopsy may be difficult because the target lesion is in the brain. Therefore, nuclear medicine imaging might be useful as one of the non-invasive methods for the diagnosis of PD. Zhao et al. performed a systematic review and meta-analysis for the accuracy of FDG PET imaging in differentiating PD from atypical *parkinsonian* syndromes (APSs) [32]. Twenty-four studies (12 studies depend on visual interpretation, and the other 12 on artificial intelligence (AI)) were assessed, and a total of 1508 PD patients and 1370 APS patients were evaluated. The pooled sensitivity, specificity, and the area under the curves (AUCs) of summary receiver operating characteristic in differentiating PD patients from APSs were 96%, 90%, and 0.98 with visual interpretation by radiologists, 93%, 90%, and 0.96 with visual interpretation supported by univariate algorithms, 87%, 91%, 0.95 with machine learning algorithms, 97%, 95%, 0.98 with deep learning algorithms, respectively. The authors described that the results suggest FDG PET has a high accuracy in differentiating PD from APSs, among which AI-assisted automatic classification performs well, with a diagnostic accuracy comparable to that of a radiology specialist, indicating that AI technology will play a key role as an important diagnostic aid tool in future clinical practice.

Primary central nervous system lymphoma (PCNSL) is an etiology of focal brain lesions in human immunodeficiency virus (HIV)-infected patients, and the incidence was reported 2% to 6%, more than 1000 times compared with the general population [33]. A robust diagnosis in the early stage is important because of the rapid progression of the disease. Some authors reported that differentiating PCNSL from other diseases, especially from toxoplasmosis in patients with HIV was difficult with X-ray, CT, and MRI [34, 35]. Yang et al. performed a systematic review and meta-analysis for diagnostic accuracy of single-photon emission computed tomography (SPECT), positron emission tomography (PET), and magnetic resonance spectroscopy (MRS) for PCNSL in patients with HIV [36]. Eventually, 18 SPECT studies (667 patients), 6 PET studies (108 patients), and 3 MRS studies (96 patients) were assessed. The pooled sensitivity and specificity in differentiating PCNSL patients from other diseases were 92% and 84% using SPECT. All six PET studies demonstrated 100% sensitivity. While four of these also achieved 100% specificity, the remaining two reported values of 91% and 75%. These lower specificities may be attributed to technological limitations, as these studies were published approximately 30 years ago. Three studies using MRS reported 71%, 50%, and 100% of sensitivity, and 83%, 60%, and 27% of specificity, respectively. The paper concluded that SPECT has good diagnostic accuracy in differentiating PCNSL from other diseases in HIV patients, and PET may have superior diagnostic accuracy than SPECT but has less supporting clinical data.

**Table 3:** Summary of diagnostic performance in neurology

Application	No. of studies	No. of patients	Pooled sensitivity	Pooled specificity
Diagnosis of Alzheimer’s disease using ¹⁸F-florbetapir amyloid PET [30]	10	682 AD / 639 controls	89.6%	87.2%
Diagnosis of Alzheimer’s disease using ¹⁸F-florbetaben amyloid PET [30]	6	682 AD / 639 controls	89.3%	87.6%
Differentiation of Parkinson’s disease from atypical Parkinsonian syndromes using FDG PET (visual interpretation) [32]	24	1508 PD / 1370 APS	96%	90%
Differentiation of Parkinson’s disease from atypical Parkinsonian syndromes using FDG PET with deep learning [32]	24	1508 PD / 1370 APS	97%	95%
Differentiation of primary CNS lymphoma from other diseases in HIV patients using SPECT imaging [36]	18	667	92%	84%
Differentiation of brain metastasis recurrence from radionecrosis using amino acid or FDG PET [37]	15	505	85%	88%

CNS, central nervous system; HIV, human immunodeficiency virus; AD, Alzheimer’s disease; PD, Parkinson’s disease; APS, atypical parkinsonian syndromes

Metastatic brain tumors are the most common brain tumors in adults, and the incidence will be 200,000 to 300,000 patients per year [37]. Stereotactic radiosurgery, which is a form of radiation therapy, has become an efficient option for patients with metastatic brain tumors [38], and radiation injury is a complication after radiation therapy. Li et al. performed a systematic review and meta-analysis for the diagnostic accuracy of amino acid (^18^F-FET, ^11^C-MET, and ^18^F-FDOPA) and FDG PET in differentiating brain metastasis recurrence from radiation necrosis after stereotactic radiosurgery [39]. Eventually, 15 studies (6 prospective and 9 retrospective studies) were assessed, and a total of 505 patients were evaluated. The pooled sensitivity, specificity, PLR, NLR, DOR and the AUCs of receiver operating characteristic of PET in differentiating brain metastasis recurrence from radio necrosis were 85%, 88%, 7.0, 0.17, 40 and 0.93. Moreover, amino acid and FDG PET had similar diagnostic accuracy. The authors concluded that amino acid and FDG PET had good diagnostic accuracy in differentiating brain metastasis recurrence from radio necrosis after radiation therapy. The pooled diagnostic accuracy of the major neurologic applications discussed above is summarized in Table 3. 

In conclusion, across oncology, cardiology, and neurology, nuclear medicine is moving toward quantitative, disease-specific imaging and therapy supported by a growing body of systematic reviews and meta-analyses. In oncology, FDG PET/CT remains foundational for response assessment and prognostication, with volumetric metrics (MTV/TLG) and metabolic response outperforming SUVmax-based approaches. In parallel, tumor-targeted tracers (PSMA, SSTR, FAPI) and expanding radioligand therapies (^177^Lu- and ^225^Ac-based) are reshaping diagnostic and therapeutic pathways. Continued generation of high-quality prospective evidence remains essential to optimize patient selection, standardize protocols, and improve clinical outcomes.
